# Fundamentals and emerging frontiers in p53-targeted drug development

**DOI:** 10.1016/j.bbrep.2026.102661

**Published:** 2026-06-04

**Authors:** Hui-Deng Long, Ning Zhang, Wen-Er Wang, Yu-Shui Ma, Fu-Xing Liu, Zi-Yu Chen, Cui-Ni Lu, Xiao-Feng Wang, Ning Han, Yue-Feng Cai, Chun Yang, Xiao-Mei Tang, Hong Yu, Hong Jiang, Da Fu, Kai-Jian Chu, Wen-Guang Wu

**Affiliations:** a Department of Pathology, The Affiliated Taizhou People's Hospital of Nanjing Medical University, Taizhou School of Clinical Medicine, Nanjing Medical University, Taizhou, Jiangsu, 225300, China; b Department of General Surgery, Ruijin Hospital, Shanghai Jiaotong University School of Medicine, Shanghai, 200025, China; c Department of Thoracic Surgery, The 905th Hospital of the Chinese People's Liberation Army Navy, Shanghai, 200052, China; d Department of General Surgery, The Fourth Hospital of Changsha, Changsha Hospital of Hunan Normal University, Changsha, Hunan, 410006, China; e Shanghai Key Laboratory of Cancer Systems Regulation and Clinical Translation, Jiading District Central Hospital Affiliated Shanghai University of Medicine & Health Science, Shanghai, 201800, China; f Department of General Surgery, The Affiliated Taizhou People's Hospital of Nanjing Medical University, Taizhou, Jiangsu, 225300, China; g Department of Biostatistics, University of Illinois Urbana-Champaign, Urbana, IL, 61801, USA; h Department of Anesthesiology and Perioperative Medicine, The First Affiliated Hospital of Nanjing Medical University, Nanjing, Jiangsu, 210029, China; i Biliary Surgical Department I, Third Affiliated Hospital of Naval Medical University, Shanghai, 200438, China; j Department of Biliary-Pancreatic Surgery, Renji Hospital, Shanghai Jiao Tong University School of Medicine, Shanghai, 200120, China

**Keywords:** p53, Tumor suppressor gene, Drug discovery, Clinical application

## Abstract

As a key tumor suppressor protein, p53 plays a central role in biological processes such as cell cycle regulation, DNA repair, apoptosis, and metabolism. However, p53 gene mutations or functional inactivation are prevalent in over 50% of human cancers, leading to tumorigenesis, development, and drug resistance, making it an important target for anticancer drug development. Currently, p53-targeted therapy faces challenges such as diverse mutation types, insufficient drug specificity, and drug resistance. This article systematically reviews the fundamental theories and cutting-edge advances in the development of p53-targeted drugs. It elaborates on the structure and function of p53 and its mutation-induced carcinogenic mechanisms, then focuses on analyzing the research history and clinical translation status of small-molecule drugs (e.g., APR-246), discusses the application prospects of gene therapy and immunotherapy strategies, and introduces emerging therapies based on CRISPR and PROTAC technologies. By integrating the latest research findings, this article aims to provide theoretical basis and directional guidance for the precise development and clinical translation of p53-targeted drugs.

## Highlights

• The p53 gene is vital for tumor suppression, regulating cell growth, death, and DNA stability.
• Recent research focuses on developing drugs to activate the p53 pathway, revolutionizing cancer treatment.
• This article explores p53 drug discovery, challenges, and future prospects, highlighting clinical potential and hurdles.

## 1 Introduction

The discovery of the *TP53* gene, which encodes the p53 protein, has significantly transformed our understanding of tumor biology and has opened new avenues for cancer treatment. As a critical regulator of the cell cycle, p53 functions as a guardian of the genome, orchestrating processes such as DNA repair, apoptosis, and cell cycle arrest in response to cellular stressors like DNA damage. When functioning correctly, p53 helps maintain genomic integrity and prevents the propagation of damaged cells, thus playing a pivotal role in cancer prevention [1,2]. However, mutations in the *TP53* gene are among the most common genetic alterations found in human cancers, occurring in over 50% of malignancies [3,4]. These mutations often lead to a loss of normal p53 function, allowing cells to evade apoptosis and proliferate uncontrollably, contributing to tumorigenesis and poor patient outcomes [5,6].

The intricate relationship between p53 and cancer has led to the exploration of therapeutic strategies aimed at restoring the function of this vital tumor suppressor. Targeting the p53 pathway has become a promising approach in cancer therapy, with various strategies being developed to reactivate mutant p53 or to inhibit the negative regulators of wild-type p53, such as MDM2 and MDMX [7,8]. The development of small-molecule inhibitors that disrupt the p53-MDM2 interaction has garnered substantial interest, as these compounds can potentially restore p53 activity in tumors harboring wild-type *TP53* [9,10]. Furthermore, recent advances in drug discovery techniques, including computational methods and high-throughput screening, have facilitated the identification of novel compounds that can selectively target p53 and its regulatory pathways [11].

In addition to small-molecule inhibitors, innovative therapeutic modalities such as gene therapy, immunotherapy, and the use of bi-specific antibodies targeting p53 have emerged as potential strategies to combat cancers characterized by p53 dysfunction [[12], [13], [14]]. For instance, gene therapy approaches aim to deliver functional copies of the *TP53* gene or to utilize CRISPR technology to correct mutations within the *TP53* locus, thereby restoring normal p53 function [15,16]. Additionally, the exploration of p53 as a target for immunotherapy has gained traction, with strategies aimed at enhancing the immune response against p53-expressing tumor cells [17].

Despite the promise of these therapeutic strategies, significant challenges remain in the clinical translation of p53-targeted therapies. The heterogeneity of *TP53* mutations and the complexity of the tumor microenvironment complicate the development of universally effective treatments [18]. Moreover, the potential for drug resistance and the need for personalized treatment approaches necessitate further research to elucidate the mechanisms underlying p53 regulation and its interactions with various cellular pathways [19].

As the field of p53 research continues to evolve, the integration of multidisciplinary approaches, including molecular biology, bioinformatics, and systems biology, will be essential in advancing our understanding of the p53 pathway and its role in cancer. This comprehensive overview of the foundational aspects and cutting-edge research surrounding p53 provides a framework for future investigations aimed at harnessing the therapeutic potential of this crucial tumor suppressor in the fight against cancer. The ongoing exploration of p53-targeted therapies holds the promise of improving treatment outcomes for patients with a wide range of malignancies, ultimately contributing to the goal of personalized cancer therapy.

## 2 The biological characteristics and functions of the *TP53* gene

### 2.1 Analysis of the structure and function of the *TP53* gene

**Fig. 1:**
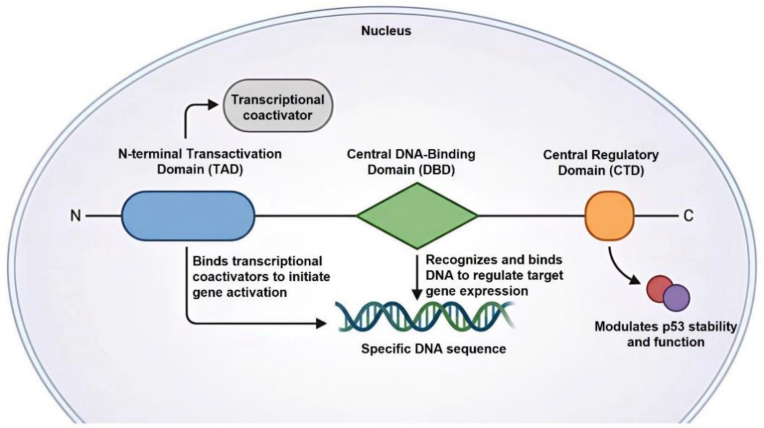
Schematic of p53 domain functions.

The *TP53* gene, located on chromosome 17p13.1, encodes the p53 protein, a crucial tumor suppressor involved in regulating the cell cycle, DNA repair, and apoptosis. Structurally, p53 is composed of several functional domains, including an N-terminal transactivation domain, a central DNA-binding domain, and a C-terminal regulatory domain (Fig. 1). The DNA-binding domain is particularly significant, as it allows p53 to bind to specific DNA sequences, activating the transcription of target genes involved in cell cycle arrest and apoptosis [20]. Mutations in the *TP53* gene, which occur in over 50% of human cancers, often lead to the production of a dysfunctional protein that loses its tumor-suppressive functions and may even gain oncogenic properties [5]. Understanding the structural aspects of p53, particularly the impact of mutations on its function, is essential for developing targeted therapies aimed at restoring its normal activity in cancer cells [3].

### 2.2 Regulation of p53 protein activity

**Fig. 2:**
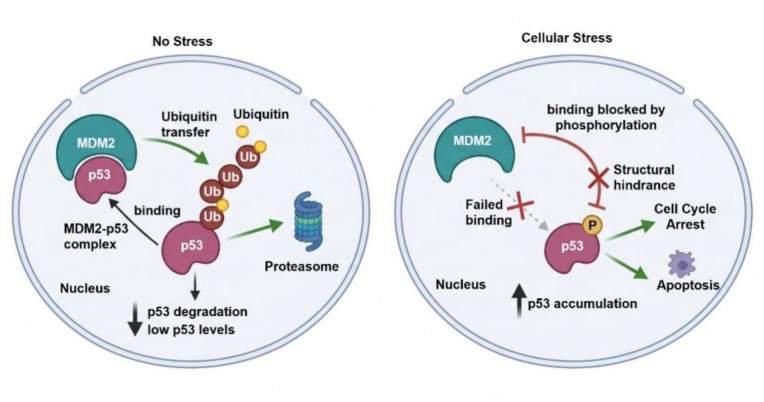
The regulatory mechanisms of p53 and MDM2.

The activity of the p53 protein is tightly regulated through various post-translational modifications, including phosphorylation, acetylation, and ubiquitination. These modifications can influence p53's stability, localization, and transcriptional activity. For instance, under non-stress conditions, MDM2 acts as an E3 ubiquitin ligase, binding to p53 to form a complex and mediating the ubiquitination modification of p53 (Fig. 2, left). Subsequently, ubiquitinated p53 is recognized and degraded by the proteasome, thereby maintaining a low basal level of p53 in the cell and preventing its overactivation [7]. In response to cellular stress, such as DNA damage, p53 undergoes phosphorylation, which disrupts its interaction with MDM2 (Fig. 2, right), leading to p53 stabilization and activation [11]. This regulatory mechanism is crucial for p53's role in mediating cellular responses to stress, including the activation of DNA repair pathways and the induction of apoptosis [9]. Therefore, targeting the p53-MDM2 interaction presents a promising therapeutic strategy for restoring p53 function in tumors with wild-type p53 [21].

### 2.3 The role of p53 in cell cycle regulation

**Fig. 3:**
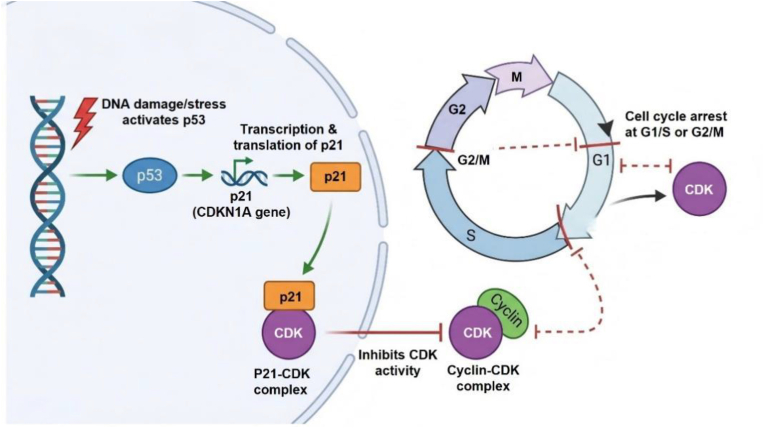
Cell cycle arrest mediated by p53-p21-CDK pathway.

p53 plays a critical role in regulating the cell cycle, primarily by inducing cell cycle arrest in response to DNA damage. Upon activation, p53 can induce the expression of various cyclin-dependent kinase inhibitors, such as p21, which inhibit cyclin-CDK complexes (Fig. 3), thereby blocking the progression of the cell cycle at the G1/S and G2/M checkpoints [20]. This function is essential for preventing the proliferation of cells with damaged DNA, allowing time for repair mechanisms to act. Moreover, p53's ability to induce cell cycle arrest is not only crucial for maintaining genomic integrity but also for facilitating apoptosis in cases where DNA damage is irreparable [11]. The intricate balance between cell cycle arrest and apoptosis is a key aspect of p53's tumor-suppressive functions, and understanding these mechanisms can aid in the development of therapies that exploit p53's regulatory roles in cancer [7].

### 2.4 The role of p53 in inducing DNA repair and apoptosis

**Fig. 4:**
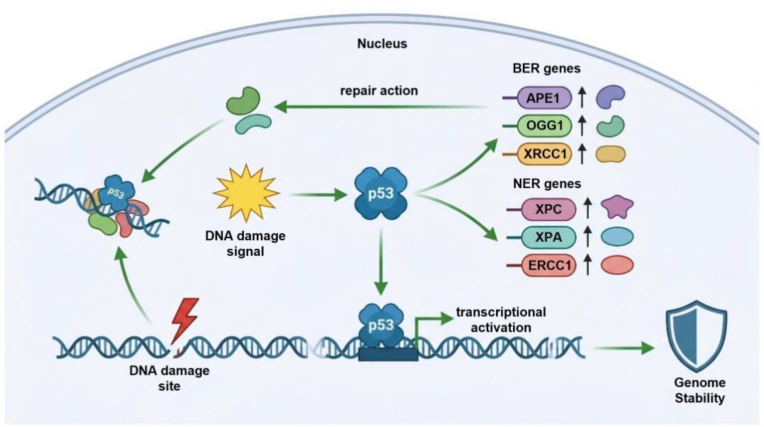
p53-mediated DNA damage response and genome stability.

p53 is often referred to as the “guardian of the genome” due to its pivotal role in maintaining genomic stability through the induction of DNA repair and apoptosis. In response to DNA damage, p53 protein is recruited to the damage site and promotes genome stability through two main pathways [22]. Primarily, p53 exerts its core function as a transcription factor, directly activating the gene expression of multiple DNA repair pathways: including base excision repair (BER)-related genes (such as *APE1*, *OGG1*, *XRCC1*) and nucleotide excision repair (NER)-related genes (such as *XPC*, *XPA*, *ERCC1*), thereby enhancing the cell's repair capacity (Fig. 4). Besides, p53 can also participate in repair actions directly at the DNA damage site independently of its transcriptional function. Through the synergistic effect of transcriptional activation and direct repair, p53 effectively maintains genome stability (Fig. 4).

**Fig. 5:**
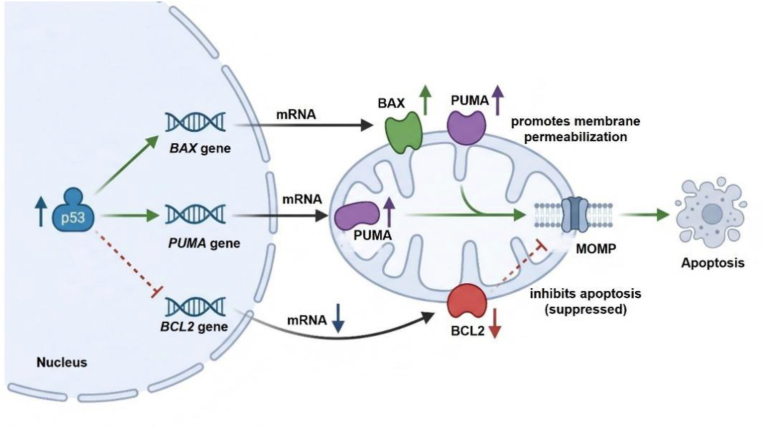
p53-mediated apoptosis regulation.

If the damage is deemed irreparable, activated p53 enters the nucleus and functions as a transcription factor [23]. On one hand, p53 significantly upregulates the transcription of pro-apoptotic genes *BAX* and *PUMA* (Fig. 5). The mRNA produced translates into proteins (BAX and PUMA) that localize to the mitochondria, promoting mitochondrial outer membrane permeabilization (MOMP). On the other hand, p53 simultaneously downregulates the expression of the anti-apoptotic gene *BCL2*, leading to a decrease in BCL2 protein levels. This dual regulation of “pro-apoptotic” and “anti-apoptotic” factors disrupts the stability of the mitochondrial membrane, ultimately driving the cell toward apoptosis. This dual role of p53 highlights its importance in preventing oncogenesis by eliminating cells carrying potential oncogenic mutations. The therapeutic implications of this function are significant, as restoring p53 activity in cancer cells can reinstate their ability to undergo apoptosis and repair DNA damage, thereby inhibiting tumor growth [7].

### 2.5 The role of p53 in inhibiting tumor angiogenesis

**Fig. 6:**
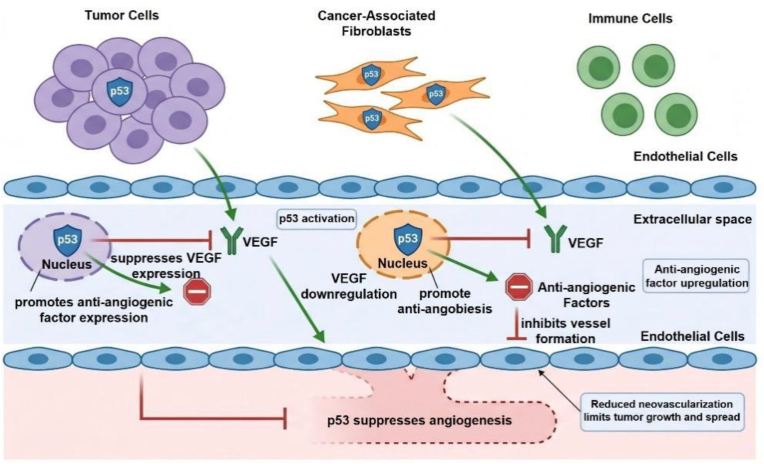
p53-mediated tumor angiogenesis suppression.

In addition to its roles in cell cycle regulation and apoptosis, p53 also exerts anti-angiogenic effects, thereby inhibiting tumor growth and metastasis. p53 can regulate the expression of various angiogenesis-related factors, including vascular endothelial growth factor (VEGF), which is crucial for the formation of new blood vessels [21]. In tumor cells and cancer-associated fibroblasts (CAFs), the activation state of p53 negatively regulates the expression of VEGF (Fig. 6). p53 not only directly inhibits the transcriptional expression of VEGF but also antagonizes angiogenic signaling by promoting the expression of multiple anti-angiogenic factors. In the extracellular space, these p53-mediated anti-angiogenic factors function to inhibit VEGF signaling. Ultimately, the effect on endothelial cells results in the inhibition of vessel formation. This reduced neovascularization effectively limits the blood supply to the tumor, thereby hindering tumor growth and spread. Furthermore, p53's influence on the tumor microenvironment extends to modulating the behavior of endothelial cells, which are essential for angiogenesis [24]. Understanding the mechanisms by which p53 inhibits angiogenesis provides valuable insights into potential therapeutic strategies aimed at enhancing p53 function to combat tumor progression [7].

### 2.6 p53 involvement in tumor cell metabolic reprogramming and microenvironment

The tumor suppressor protein p53 is a pivotal player in cellular responses to stress, particularly in the context of tumorigenesis. Its role extends beyond merely regulating the cell cycle and apoptosis; it significantly influences metabolic pathways that are crucial for tumor cell survival and proliferation. Cancer cells often undergo metabolic reprogramming to support their rapid growth and survival in hostile environments. This phenomenon, commonly referred to as the Warburg effect, involves a shift from oxidative phosphorylation to aerobic glycolysis, allowing cancer cells to generate energy and biosynthetic precursors more efficiently, even in the presence of oxygen [25]. p53 contributes to this metabolic regulation by promoting oxidative phosphorylation and inhibiting glycolysis, thus counteracting the metabolic adaptations characteristic of cancer cells [26].

Moreover, p53's involvement in metabolic reprogramming is not limited to its transcriptional regulatory functions. It also interacts with various metabolic pathways, including lipid and amino acid metabolism, to maintain cellular homeostasis and prevent tumorigenesis. For instance, p53 has been shown to regulate lipid metabolism, which is critical for membrane synthesis and energy storage in cancer cells [27]. Additionally, p53 influences the pentose phosphate pathway, which is essential for nucleotide synthesis and cellular antioxidant defense, thereby protecting cells from oxidative stress [28].

The tumor microenvironment also plays a crucial role in shaping the metabolic landscape of cancer cells. The interaction between p53 and the tumor microenvironment can lead to significant changes in tumor cell metabolism. For example, in hypoxic conditions, p53 can activate genes that promote angiogenesis and metabolic adaptation, allowing tumors to thrive despite limited oxygen and nutrient availability [29]. Furthermore, *TP53* mutations, which are prevalent in many cancers, can lead to a loss of these regulatory functions, resulting in enhanced glycolytic metabolism and increased tumor aggressiveness [30].

p53 is integral to the metabolic reprogramming of tumor cells, influencing both intrinsic metabolic pathways and interactions with the tumor microenvironment. Its ability to regulate these processes underscores its critical role in tumor suppression and highlights the potential for therapeutic strategies aimed at restoring p53 function to counteract cancer progression.

## 3 Mechanisms of p53 inactivation in cancer: mutation and negative regulation

### 3.1 Types of *TP53* gene mutations

The mutation spectrum of the *TP53* gene is highly heterogeneous, with mutation types primarily including missense mutations, nonsense mutations, frameshift mutations, and splice site mutations, among which missense mutations are the most common (Table 1), accounting for approximately 75% of all *TP53* mutations [14,31]. These missense mutations mainly occur in exons encoding the DNA-binding domain, leading to single amino acid substitutions that profoundly affect the structure and function of the p53 protein [32,33]. Based on their impact on protein structure, missense mutations can be roughly divided into two categories: one is DNA contact mutations, such as R248 and R273, which directly disrupt the binding ability of the p53 protein to DNA target sequences, resulting in loss of p53 inhibitory function (LOF); the other is structural mutations, such as R175 and G245, which indirectly affect DNA binding function by disrupting the zinc ion coordination or overall folding stability of the core domain of the p53 protein [33,34]. For example, in-depth studies of the R248 site have found that when arginine (R) is replaced by different amino acids such as tryptophan (W), glycine (G), or proline (P), it destabilizes the p53-DNA complex in different ways, reduces its DNA binding affinity, and may promote gain-of-function (GOF) phenotypes [35,36]. Additionally, although splice site mutations account for a relatively small proportion (approximately 7%), their functional and clinical consequences have long been overlooked. Studies have shown that such mutations can lead to reduced *TP5*3 mRNA levels, abnormal protein expression, and trigger unique transcriptomic phenotypes independent of tumor type, affecting genomic stability and patient prognosis [37,38].

**Table 1:** Major types, structural characteristics, and functional impacts of TP53 mutations.

Mutation Type	Typical Structural Features/Location	Core Functional Impact	Association with Cancer Types
Missense Mutation (Most common, ∼75%)	DNA-binding domain (Hotspots: R175, R248, R273, etc.)	Loss of Function: Conformational change, unable to bind DNA; Dominant Negative Effect: Inhibits the function of wild-type p53; Gain of Function: Some mutants (e.g., R175H) acquire new pro-oncogenic functions (invasion, metabolic reprogramming).	Pan-cancer (Ovarian cancer, breast cancer, lung cancer, colorectal cancer); R249S is strongly associated with liver cancer (aflatoxin-related).
Nonsense Mutation	Anywhere in the coding region (Generates a premature termination codon)	Loss of Function: Triggers NMD degradation or produces a nonfunctional truncated protein.	Li-Fraumeni syndrome, sporadic breast cancer, hematologic tumors.
Frameshift Mutation	Insertions/Deletions (Indels) (Mostly located in the DNA-binding domain)	Loss of Function: Altered reading frame, producing abnormal truncated proteins.	Colorectal cancer, gastric cancer (microsatellite instability-high type).
Splice Site Mutation	Intron boundaries (Donor/acceptor sites)	Loss of Function: Aberrant mRNA splicing, producing nonfunctional isoforms.	Various hereditary and sporadic tumors.
Non-coding Region Mutation	Promoter/Regulatory regions	Dysregulated Expression: Affects transcription efficiency or protein expression levels.	Relatively rare, reported in some hematologic tumors.

### 3.2 Gain-of-function (GOF) of mutant p53

The functional consequences of *TP53* gene mutations extend far beyond simple loss of tumor suppressor function (LOF). A large body of research indicates that many mutant p53 proteins acquire new oncogenic functions, known as GOF [14,39]. GOF activity enables mutant p53 proteins to drive tumor progression through various mechanisms, such as promoting genomic instability, reprogramming cell metabolism, enhancing cancer invasion and metastasis, and remodeling the tumor microenvironment [40,41]. In adenocarcinoma of the lung, *TP53* mutations cause tumor cells to lose alveolar characteristics, upregulate gene expression programs associated with high proliferation and entropy, and shape a multicellular tumor niche composed of SPP1^+^ macrophages and collagen-expressing fibroblasts, which is characterized by anoxia and pro-metastatic properties [42]. In head and neck squamous cell carcinoma, *TP53* mutations are associated with suppressed immune features, affecting immune cell infiltration and the expression of immune-related genes [43,44]. Furthermore, mutant p53 can also form non-functional complexes with residual wild-type p53 or other p53 family members (such as p63 and p73) within cells through a dominant-negative effect, further inhibiting its tumor suppressor activity [45,46]. This GOF activity makes therapeutic strategies targeting mutant p53 particularly complex and necessary.

### 3.3 Negative regulatory mechanism of MDM2/MDMX on p53

In *TP53* wild-type tumors, the tumor suppressor activity of p53 is primarily regulated by its key negative regulators MDM2 and MDMX through precise mechanisms. MDM2 is a direct transcriptional target gene of p53, forming a classic negative feedback loop: when p53 is activated, it promotes the transcriptional expression of MDM2; subsequently, MDM2 protein, through its E3 ubiquitin ligase activity, ubiquitinates p53, leading to its degradation via the proteasome pathway, thereby maintaining p53 activity and levels within a physiological range [47,48]. MDMX shares structural similarity with MDM2 but lacks effective E3 ubiquitin ligase activity. It primarily inhibits p53 activity by directly binding to its transcriptional activation domain and forms heterodimers with MDM2 to coordinately regulate p53. MDMX stabilizes MDM2 and then recruits ubiquitin-conjugating enzymes (e.g., UbcH5c), thereby enhancing the efficiency of MDM2-mediated p53 ubiquitination and further accelerating p53 degradation [49,50]. However, in various tumors, amplification or overexpression of the MDM2 gene is one of the main mechanisms leading to the suppression of wild-type p53 function, thereby promoting tumorigenesis. For example, in certain sarcomas, breast neoplasms, and gliomas, MDM2 overexpression prevents effective accumulation and tumor-suppressive function of p53 protein, even in the presence of an intact wild-type TP53 gene. In addition to genetic alterations, single nucleotide polymorphisms (SNPs) in MDM2, such as SNP309 (T > G), may weaken p53 pathway function by increasing MDM2 expression, thereby affecting individual susceptibility to cancer and treatment response [51]. Therefore, targeting the MDM2-p53 interaction using small molecule inhibitors such as Nutlin-3a to block MDM2 binding to p53, thereby stabilizing and activating p53, has become an important therapeutic strategy for treating tumors harboring wild-type *TP53* [51,52]. However, MDM2 inhibitors may face resistance challenges in clinical applications, partly due to compensatory upregulation of MDMX or activation of alternative signaling pathways.

### 3.4 Impact of p53 mutations on drug development

The mutational status of p53 profoundly influences the efficacy and development strategies of anticancer drugs. First, mutation-induced conformational changes in p53 typically weaken its interaction with the E3 ubiquitin ligase MDM2, rendering traditional MDM2 inhibitors (designed to stabilize wild-type p53) less effective against tumors carrying mutant p53 [53]. Second, different mutation types exhibit significant differences in drug sensitivity. For instance, in non-small cell lung cancer, patients with *TP53* mutations (particularly in exons 6 and 7) show significantly lower response rates and progression-free survival to the EGFR-TKI gefitinib compared to *TP53* wild-type patients [54,55]. This highlights the importance of developing conformation-specific p53 reactivators; for example, the natural compound celastrol has been shown to induce heat shock protein 70 (HSP70) transcription, promoting MDM2-mediated proteasomal degradation of TP53R175H and TP53Y220C mutant proteins, thereby inhibiting tumor growth [56,57]. Furthermore, the GOF activity of mutant p53 itself has become a promising new drug target. Dual-specific antibodies targeting neoantigens derived from *TP53* mutations (e.g., the most common R175H mutation) have shown potential in preclinical models to activate T cell-mediated specific killing of cancer cells [57]. Additionally, leveraging genomic instability caused by mutations in *TP53* or ATM, synthetic lethality strategies targeting DNA damage response pathway inhibitors (e.g., ATR, CHK1, WEE1 inhibitors) have demonstrated therapeutic promise [58,59]. These advances underscore the critical importance of understanding the functional consequences of p53 mutations for developing precise, mutation-specific therapeutic strategies.

## 4 Research progress on classic p53-targeted small molecule drugs

### 4.1 Small molecule drugs that restore mutant p53 function

APR-246 (PRIMA-1MET) is the first mutant p53 reactivator to enter clinical trials, with a unique mechanism of action (Table 2). This drug is converted in vivo into the active metabolite methylene quinuclidinone (MQ), which covalently binds to cysteine residues in mutant p53 protein, thereby inducing the refolding of mutant p53 into a wild-type conformation and restoring its transcriptional activation function and pro-apoptotic ability [59]. Preclinical studies have shown that APR-246 effectively induces apoptosis in a variety of tumor cells carrying p53 mutations. For example, in esophageal squamous cell carcinoma, APR-246 induces reactive oxygen species (ROS) production, upregulates p73 and Noxa signaling pathways, thereby specifically killing tumor cells with p53 missense mutations [60]. Furthermore, when combined with chemotherapeutic drugs such as 5-fluorouracil (5-FU), APR-246 exhibits synergistic effects in pancreatic cancer cell lines, significantly enhancing the killing effect on tumor cells [61,62]. In hematological malignancies, the combination of APR-246 with azacitidine has shown an overall response rate of up to 71% and a complete response rate of 44% in patients with TP53-mutated myelodysplastic syndromes and acute myeloid leukemia (AML) [63]. However, the clinical application of APR-246 also faces challenges. Studies have found that the sensitivity of tumor cells to APR-246 depends not only on TP53 mutation status but is also closely related to SLC7A11 expression levels, with SLC7A11 being a better predictive biomarker for efficacy than *TP53* mutation status [64]. Additionally, the development of drug resistance and optimization of drug bioavailability remain key obstacles in its clinical translation [65].

**Table 2:** Overview of Therapeutic Strategies and Candidate Drugs Targeting the p53 Pathway.

Therapeutic Strategy	Representative Drug/Candidate	Mechanism of Action	Stage of Development	Key Clinical Challenges and Prospects
Restore Mutant p53 Conformation and Function	APR-246 (Eprenetapopt)	Covalently binds to mutant p53 (e.g., R175H), refolding it to a wild-type conformation, restoring transcriptional and pro-apoptotic function.	Phase III (MDS/AML)	Challenges: Limited efficacy for non-R175H mutations; Phase III trial failed to meet primary endpoint (CR rate), development encountering hurdles. Prospects: The first mutant p53 reactivator to reach late-stage trials, providing proof of concept.
Inhibit MDM2-p53 Interaction	Idasanutlin (RG7388)	Highly selective MDM2 antagonist, blocks MDM2-mediated p53 degradation, activating the wild-type p53 pathway.	Phase III (AML, etc.)	Challenges: Strictly dependent on a wild-type TP53​ background; monotherapy can cause thrombocytopenia and gastrointestinal toxicity. Prospects: Combination therapy with Bcl-2 inhibitors or chemotherapy is a key direction to improve efficacy.
Target Mutant p53 for Degradation	Hsp90 Inhibitor (e.g., Ganetespib)	Inhibits the chaperone Hsp90, leading to ubiquitination and degradation of its client proteins, including mutant p53.	Phase II	Challenges: Broad client protein profile leads to significant systemic toxicity; ineffective in tumors with wild-type p53. Outlook: Development interest has waned due to narrow therapeutic window.
Synthetic Lethality Strategy (p53 LOF context)	WEE1 Inhibitor (e.g., Adavosertib)	p53-deficient cells rely on the G2/M checkpoint. Inhibiting WEE1 kinase leads to DNA damage accumulation and “mitotic catastrophe."	Phase II/III	Challenges: Requires precise enrichment of p53-mutant patients; combination with chemotherapy may exacerbate myelosuppression. Prospects: High potential in cancers with high TP53 mutation rates like ovarian and pancreatic cancer.
	PARP Inhibitor (e.g., Olaparib)	p53 loss is often associated with Homologous Recombination (HR) defect. PARPi induces synthetic lethality in an HR-deficient (HRD) context.	Approved	Challenges: Not all p53 mutations confer HRD, requiring patient selection using biomarkers like BRCA status.
Emerging Strategies (GOF/Liquid-Liquid Phase Separation)	ReACp53	Cell-penetrating peptide that inhibits the formation of amyloid aggregates by mutant p53, disrupting its Gain-of-Function (GOF) activity.	Preclinical	Challenges: Delivery efficiency and in vivo stability of peptide drugs are major bottlenecks. Prospects: Provides a novel concept for targeting the “undruggable” p53 aggregates.
Gene Therapy	Gendicine™ (Recombinant human p53 adenovirus)	Delivers the wild-type p53 gene into tumor cells via an adenoviral vector, restoring p53 expression.	Approved in China (Head and neck cancer)	Challenges: Limited application due to local injection requirement; high immunogenicity and ineffective against metastases. Outlook: The world's first commercially approved gene therapy product, but its use remains limited.

As a pioneer in p53-targeted drug development, the clinical research progress of APR-246 provides an important window for evaluating the potential and challenges of such drugs. This drug has entered several phase I/II clinical trials, such as the NCT03072043 study for AML and the NCT03745716 study for solid tumors including ovarian and prostate cancers [66,67]. However, the clinical application of APR-246 faces multiple limitations. First, the drug currently requires intravenous administration, leading to low bioavailability, which causes inconvenience for long-term patient treatment and may affect the effective drug concentration in tumor tissues [68,69]. Second, APR-246 shows poor efficacy against certain specific p53 mutation types (e.g., R273H), indicating mutation-specific efficacy differences [70]. This mutation selectivity limits its potential as a broad-spectrum p53 reactivator. A more fundamental challenge lies in tumor heterogeneity, where the same tumor may contain both sensitive and insensitive cell subpopulations to APR-246, allowing some cells to escape therapeutic pressure and ultimately leading to disease progression or relapse [71,72].

### 4.2 Small molecule drugs that inhibit MDM2-p53 interaction

Nutlin-3a is a classic MDM2 inhibitor that mimics the transactivation domain (TAD) of p53, competitively binding to MDM2 protein, thereby blocking MDM2's negative regulation of p53, stabilizing and activating wild-type p53 function [73]. In mesothelioma cells, Nutlin-3a preferentially inhibits the growth of wild-type TP53 cells and upregulates p53 protein levels without causing DNA damage [74]. Based on the success of Nutlin-3a, second-generation MDM2 inhibitors such as RG7112 and Idasanutlin (RG7388) have been developed, with higher affinity and better pharmacokinetic properties [75]. Idasanutlin (Table 2) shows potent anti-leukemic activity in preclinical models of acute lymphoblastic leukemia (ALL), effectively reducing the viability of various ALL cell lines and inducing upregulation of pro-apoptotic gene expression profiles [76]. However, the efficacy of MDM2 inhibitors in solid tumors is limited, possibly due to the complex microenvironment of solid tumors and the heterogeneity of the p53 signaling pathway [77]. Additionally, MDM2 inhibitors are often accompanied by dose-dependent hematological toxicities in clinical applications, such as thrombocytopenia and neutropenia, which limit their single-agent dosage and efficacy [9,53]. Therefore, combination therapy strategies have become an important direction to overcome these limitations. For example, the combination of Idasanutlin with the BCLXL/BCL2 inhibitor navitoclax shows synergistic lethality in ALL models, providing new treatment ideas for high-risk and relapsed patients [78].

### 4.3 Drugs targeting p53 post-translational modifications

The transcriptional activity and stability of p53 are precisely regulated by various post-translational modifications, with acetylation and phosphorylation being two key modifications. Histone deacetylase inhibitors (HDACi) such as SAHA (Vorinostat) enhance p53 acetylation levels by inhibiting HDAC activity, thereby restoring or enhancing its transcriptional activity [79]. In *TP53*-mutated multiple myeloma cells, SAHA treatment upregulates CDKN1A (p21) gene expression in a p53-independent manner and induces apoptosis, demonstrating its potential as a broad-spectrum anticancer drug [80]. Furthermore, the combination of HDAC inhibitors with APR-246 shows synergistic effects in neuroblastoma models, significantly enhancing APR-246's anti-tumor activity by jointly elevating ROS levels [81]. On the other hand, phosphatase inhibitors such as Fostamatinib, by blocking MDM2-mediated p53 dephosphorylation, can prolong the half-life of p53 protein, thereby stabilizing its function [82]. Studies show that Fostamatinib can bind to ANKRD22 protein, which is highly expressed in pancreatic cancer, and may inhibit tumor growth by suppressing its function [83]. These drugs targeting p53 post-translational modifications are often used as part of combination therapy strategies, combined with other p53-targeted drugs (such as MDM2 inhibitors or mutant p53 reactivators), aiming to enhance anti-tumor efficacy and overcome potential drug resistance in tumor cells through multi-pathway synergy [84].

### 4.4 Mutant p53 degradation inducers

Promoting the degradation of mutant p53 protein is another effective strategy to inhibit tumor progression by eliminating the oncogenic activity of GOF mutant p53 [85]. The advantage of this strategy is that it does not rely on restoring p53 function but directly eliminates the oncogenic effects of the mutant protein [13]. Over 50% of cancers harbor TP53 mutations, and highly stable mutant p53 protein drives tumor initiation and progression through GOF [31]. The abnormal accumulation of mutant p53 is mainly regulated by molecular chaperones (e.g., Hsp40, Hsp70, Hsp90) and other biomolecules (e.g., TRIM21, BAG2, and Stat3) [40]. Therefore, targeting these factors that stabilize mutant p53 to induce its degradation via the ubiquitin-proteasome pathway has become a feasible therapeutic direction [86]. Heat shock protein (e.g., Hsp90) inhibitors and histone deacetylase (HDAC) inhibitors can induce mutant p53 degradation and have shown anti-tumor activity in preclinical models [87]. For example, the molecular chaperone Hsp90 plays a key role in maintaining the stability and function of many oncoproteins, including certain mutant p53 [88]. The Hsp90 inhibitor Ganetespib (Table 2) can interfere with its interaction with client proteins, leading to ubiquitination and subsequent proteasomal degradation of mutant p53 [89]. Similarly, HDAC inhibitors, by altering histone acetylation status and affecting acetylation of non-histone proteins (including p53), can promote mutant p53 degradation or alter its function [90]. Additionally, studies have found that Valosin-containing protein (VCP), as an upstream regulator of the BAP1 tumor suppressor protein, can bind to BAP1 and promote its degradation via the ubiquitin-proteasome pathway, thereby promoting cholangiocarcinoma development; conversely, VCP inhibitors can inhibit cancer cell growth and promote apoptosis by blocking BAP1 ubiquitination degradation [91]. Although this is an example targeting other tumor suppressor proteins, it illustrates a universal strategy for regulating key protein stability by intervening in the ubiquitination degradation mechanism. Therefore, developing small molecules that specifically promote mutant p53 degradation provides another highly promising approach for treating cancers carrying TP53 mutations.

## 5 Emerging p53-targeted therapeutic strategies

### 5.1 CRISPR/Cas9-based gene editing therapy

CRISPR/Cas9 technology provides an unprecedented precise tool for directly repairing or knocking out mutant *TP53* genes, aiming to restore the tumor suppressive function of wild-type p53. This technology can design specific guide RNAs to direct Cas9 nuclease to generate double-strand breaks at mutation sites, followed by introducing correct DNA sequences through homologous recombination repair mechanisms, thereby correcting p53 mutations [92]. Additionally, using CRISPR-Cas9 to knock out mutant p53 genes can eliminate their dominant-negative effects and gain-of-function activities. Studies have shown that in CRISPR-Cas9-edited lung adenocarcinoma cells, deletion of key regions of p53 alters its multidimensional structure, thereby affecting cell sensitivity to PI3K inhibitors [93,94]. However, optimization of in vivo delivery systems such as adeno-associated virus and lipid nanoparticles is crucial for improving gene editing efficiency, and tumor regression effects have been observed in animal models [95]. Despite promising prospects, this strategy still faces major challenges including off-target effects, immunogenicity, and delivery efficiency, and is currently mainly in preclinical research stages [96]. Notably, CRISPR-Cas9 editing itself may induce p53-mediated stress responses and cell cycle arrest, but no significant p53 activation has been observed in gene-edited animal models [97]. Furthermore, achieving efficient and precise genome editing in human induced pluripotent stem cells using CRISPR technology requires combining p53 inhibition and pro-survival small molecules to improve homologous recombination rates [98].

### 5.2 PROTAC technology for targeted degradation of mutant p53

Proteolysis-targeting chimeras (PROTAC) technology provides a revolutionary strategy for targeting “undruggable” mutant p53 proteins. PROTAC is a bifunctional molecule that binds to the target protein (e.g., mutant p53) on one end and recruits E3 ubiquitin ligase on the other, thereby inducing ubiquitination and proteasomal degradation of the target protein [99]. For example, researchers developed a high-performance DNA aptamer through iterative molecular docking-guided SELEX methods, and based on this, designed a selective p53-R175H degrader dp53 m, which showed significant anti-tumor activity both in vivo and in vitro, and synergistically enhanced Cisplatin sensitivity [100]. Additionally, the MDM2-targeting PROTAC molecule MS3227 degrades MDM2 by recruiting VHL E3 ligase, thereby activating the p53 pathway, and demonstrated stronger anti-leukemic activity than traditional inhibitors in acute leukemia models [101]. PROTAC technology has the potential for catalytic activity, high selectivity, and overcoming drug resistance. For instance, degradation of LZK kinase can simultaneously reduce levels of c-MYC and gain-of-function p53, inhibiting the growth of head and neck squamous cell carcinoma [102]. However, this technology still requires optimization of pharmacokinetic properties, reduction of potential toxicity, and resolution of off-target degradation issues [103]. Furthermore, MDM2-based PROTACs show great potential in cancer therapy through a dual mechanism (degrading target proteins and activating p53) [104].

### 5.3 p53-dependent immunotherapy strategies

Restoring p53 function can reshape the tumor immune microenvironment and enhance the efficacy of immunotherapy. Activation of p53 upregulates the expression of MHC class I molecules on the surface of tumor cells and regulates immune checkpoint levels such as PD-L1, thereby enhancing T cell recognition and killing of tumor cells [105]. Studies have shown that p53 loss promotes PD-L1 expression and reduces CD8^+^ T cell infiltration, while restoring p53 activity reverses this process [106]. Therefore, combining p53-targeted drugs with immune checkpoint inhibitors (e.g., anti-PD-1/PD-L1) shows synergistic anti-tumor effects in preclinical models. For example, MDM2 inhibitors, by inducing p53, increase the production of IL15 and MHC-II, thereby enhancing the efficacy of anti-PD-1 immunotherapy [107]. Additionally, triple therapy using adenovirus vector-delivered p53 gene (Ad-p53) combined with CD122/132 agonists and anti-PD-1 antibodies achieved complete tumor regression in melanoma models and produced abscopal effects [108]. p53 mutant peptide vaccines (e.g., p53-SLP) are also undergoing clinical trials, aiming to activate specific T cell immunity [13]. Notably, TP53 mutation status itself can serve as a predictive biomarker for the efficacy of immune checkpoint inhibitors. For example, in pulmonary sarcomatoid carcinoma, TP53 mutations are associated with longer progression-free survival [53].

### 5.4 p53 aggregation inhibitor-based therapy

Abnormal aggregation of mutant p53 protein is an important mechanism leading to its gain-of-function and promotion of tumor progression. The hydrophobic core of mutant p53 protein is exposed, leading to the formation of amorphous aggregates. These aggregates not only inactivate wild-type p53 through dominant-negative effects but also acquire new functions that promote tumor metastasis and drug resistance [53]. The small molecule ReACp53 effectively inhibits aggregation by binding to the hydrophobic core of mutant p53 and restores partial wild-type p53 function. In breast cancer and pancreatic cancer models, ReACp53 induces apoptosis and inhibits tumor metastasis, showing synergistic effects with chemotherapeutic drugs. This strategy directly targets the unique mechanism of p53 aggregation, providing new ideas for treating tumors carrying p53 mutations. Additionally, other small molecules such as PRIMA-1MET can exert anti-tumor effects by restoring the wild-type conformation of mutant p53, showing cytotoxic effects in chronic lymphocytic leukemia cells and correlating with reduced p53 protein levels [109]. For specific mutations such as Y220C, the small molecule reactivator rezatapopt (PC14586) restores DNA binding ability and transcriptional activity by correcting the conformation of the mutant protein, showing preliminary efficacy in clinical trials [110]. These studies collectively indicate that targeting p53 aggregation and conformational abnormalities is a highly promising therapeutic direction.

### 5.5 Intervention targeting liquid-liquid phase separation processes

Research has found that mutant p53 (mutp53) can undergo liquid-liquid phase separation (LLPS), forming “mesoscopic protein-enriched clusters” approximately 40 nm in size. Their formation is primarily driven by ordered domains of p53 (such as DBD). Specific oncogenic mutations (e.g., M237I and R249S) accelerate this process by disrupting local protein structure and enhancing intermolecular interactions [[111], [112], [113], [114]]. This phase separation provides a concentrated precursor for subsequent amyloid fibril formation. In cells, the phase separation of mutp53 is associated with its oncogenic function, as observed in lung squamous cell carcinoma patient-derived organoids (PDOs) where related pathway activation was noted [115,116]. This suggests that mutp53 droplets may serve as platforms for aberrantly recruiting signaling molecules, driving tumor progression. Therefore, understanding this mechanism provides new targets for intervening in its function.

LLPS is the initial step in the pathological aggregation of proteins like mutp53, and intervening in this process represents an upstream therapeutic strategy. Changes in intracellular ion concentration, pH, and redox state can affect protein phase separation. For example, zinc ions (Zn^2+^) are crucial for maintaining the correct conformation of p53, and abnormal concentrations may increase the propensity for p53 misfolding and aggregation [111,116,117]. Therefore, regulating intracellular Zn^2+^ concentration may help maintain mutp53 conformational homeostasis and prevent pathological phase separation. Similarly, redox imbalance (e.g., elevated ROS) is a significant factor driving phase separation and aggregation, and scavenging ROS or inhibiting its production can alleviate p53-related cellular damage [118,119]. Developing compounds that specifically bind to mutp53 droplets is another direct strategy, which can prevent droplet maturation, solidification, or promote dissolution, thereby blocking the pathological process at an early stage. Strategies for dissolving protein droplets in neurodegenerative diseases may be applicable to cancer therapy, as mutp53 aggregation is associated with poor prognosis [115,120]. Furthermore, modulating the molecular chaperone network is key to maintaining protein conformational homeostasis and preventing pathological phase separation. Heat shock proteins (HSPs) such as HSP70 and HSP90 play central roles in protein folding, transport, and degradation. Studies show that heat shock transcription factor 1 (HSF1) regulates the expression of HSP70, HSP27, etc., and its deficiency activates the ROS/p53/p21 pathway, accelerating cellular senescence and increasing oxidative stress sensitivity [121,122]. This suggests that activating HSF1 or enhancing HSP activity may help maintain mutp53 in a soluble state, preventing phase separation and aggregation. Conversely, inhibiting the activity of specific molecular chaperones associated with abnormal mutp53 aggregation may also become a therapeutic strategy. By intervening in the initial event of LLPS from multiple angles, it is hoped to curb the formation of mutp53 amyloid aggregates at the source, providing new avenues for cancer treatment.

## 6 Emerging frontiers: gene therapy and immunotherapy

### 6.1 p53 gene therapy and gene editing

p53 gene therapy and gene editing are frontier strategies targeting p53-mutated tumors. Among these, p53 gene replacement therapy based on Adenovirus vector has achieved milestone progress. For example, recombinant human p53 adenovirus injection (Gendicine) was approved in China in 2003 for the treatment of Head and neck squamous cell carcinoma, becoming the world's first approved gene therapy product [123]. However, the efficacy of this therapy is limited by issues such as delivery efficiency, pre-existing immune responses, and tumor heterogeneity [124]. To overcome these limitations, CRISPR-Cas9 gene editing technology has been used to directly repair p53 mutations or knock out mutant p53, showing great potential in preclinical models [97]. For instance, knocking out TP53 and CDKN2A in a gastroesophageal junction organoid model using CRISPR-Cas9 successfully induced tumor formation, providing a powerful tool for studying early tumorigenesis mechanisms [125]. Additionally, base editing and prime editing technologies can precisely correct point mutations without generating DNA double-strand breaks, offering more precise therapeutic possibilities for p53-mutated tumors [126]. These emerging technologies are expected to overcome the bottlenecks of traditional gene therapy and propel p53-targeted therapy into a new phase.

### 6.2 p53 and immune microenvironment regulation

The functional status of p53 profoundly influences the tumor immune microenvironment. Restoring wild-type p53 function can significantly enhance tumor immunogenicity by upregulating the expression of major histocompatibility complex class I (MHC-I) molecules and various chemokines, promoting the infiltration and activation of cytotoxic T cells [127]. Conversely, the gain-of-function (GOF) activity of mutant p53 protein can suppress anti-tumor immune responses, for example, by downregulating the expression of programmed death-ligand 1 (PD-L1) or recruiting immunosuppressive cells (such as regulatory T cells and tumor-associated macrophages) to create an immunosuppressive microenvironment [128]. Studies indicate that p53 mutation status is closely related to the efficacy of immune checkpoint inhibitors. For example, in non-small cell lung cancer, patients with co-mutations of KRAS and TP53 who receive PD-1 inhibitors combined with chemotherapy have significantly longer progression-free survival than those without such co-mutations [129]. Therefore, the combination of p53-targeted drugs and immune checkpoint inhibitors (such as anti-PD-1/PD-L1 antibodies) shows synergistic anti-tumor effects in preclinical models, aiming to achieve more durable tumor control by simultaneously restoring p53 function and relieving immune suppression [130].

### 6.3 p53 vaccines and adoptive cell transfer therapy

Immunotherapeutic strategies based on p53, including vaccines and Adoptive cell transfer therapy, provide new avenues for targeting p53-mutated tumors. p53 mutation peptide vaccines aim to induce specific T cell immunity by targeting abnormal peptides (neoantigens) generated by mutant p53 protein. Preclinical studies have confirmed that point-mutated p53 peptides can induce peptide-specific cytotoxic T lymphocyte (CTL) responses, providing experimental evidence for clinical tumor immunotherapy [131]. Additionally, p53-based dendritic cell (DC) vaccines activate anti-tumor immune responses by presenting p53 antigens to the immune system. For example, transfecting the p53 gene into CD34^+^ hematopoietic stem cell-derived DCs can induce specific CTL responses, effectively killing hepatocellular carcinoma cells expressing p53 antigens [132]. In the field of Adoptive cell transfer therapy, Chimeric antigen receptor T cell (CAR-T cell) therapy targeting p53 mutation-related antigens (such as mutant peptides presented by MHC-I molecules) is an emerging research direction. Although CAR-T cell therapy has achieved significant success in hematological malignancies, it still faces challenges in solid tumors, such as difficulty in tumor infiltration, antigen escape, and an immunosuppressive microenvironment [133]. In the future, optimizing CAR-T cells through Gene editing technology, such as enhancing their persistence and tumor homing ability, may improve their efficacy in p53-mutated solid tumors [134].

## 7 Synthetic lethality strategy and p53-deficient tumors

### 7.1 Principles of synthetic lethality and target selection

Synthetic lethality refers to a biological phenomenon where the inactivation of a single gene in two genes or pathways does not lead to cell death, but simultaneous inactivation of both genes triggers cell death [135]. In the field of tumor therapy, this principle is cleverly applied to target p53-deficient tumor cells. Due to the loss of p53 function leading to failure of the G1/S cell cycle checkpoint, tumor cells are highly dependent on the G2/M checkpoint (regulated by ATR/CHK1/WEE1 kinases) to maintain genomic stability [136]. Therefore, inhibiting WEE1 kinase (e.g., adavosertib) can force p53-deficient cells into mitotic catastrophe, achieving selective killing. Additionally, ATR and CHK1 inhibitors also exhibit synthetic lethality potential in TP53-mutant tumors by blocking DNA damage repair pathways, inducing replication stress and apoptosis [137].

Beyond cell cycle checkpoints, DNA damage repair pathways are also important targets for synthetic lethality. The successful application of PARP1 inhibitors in BRCA1/2-mutant tumors is a classic example of the synthetic lethality concept [138]. In tumors with p53 deficiency and BRCA1/2 mutations, PARP inhibition leads to accumulation of DNA single-strand breaks, subsequently causing replication fork collapse and double-strand breaks, ultimately resulting in cell death [139]. CRISPR whole-genome screening has identified multiple genes that are synthetic lethal with p53, including PLK1, CDK1, and TOP2A [140]. Among these, PLK1, a mitotic kinase, is overactive in p53-deficient cells, and its inhibition can induce mitotic catastrophe [141]. Aurora A kinase has also been confirmed as a synthetic lethal partner of p53, and its inhibition can specifically kill p53-mutant cells by regulating G2/M transition and spindle assembly [141].

### 7.2 Representative drugs and preclinical evidence

The WEE1 inhibitor adavosertib (AZD1775) has shown significant preclinical activity in p53-mutant tumors. WEE1 is a key kinase of the G2/M cell cycle checkpoint, and in tumor cells with p53 functional deficiency, cells rely more on the G2/M checkpoint to repair DNA damage due to the loss of the G1/S checkpoint [136]. Adavosertib forces p53-mutant cells into mitosis by inhibiting WEE1, leading to DNA damage accumulation, abnormal mitosis, and apoptosis [142]. In p53-mutant ovarian cancer, pancreatic cancer, and colorectal cancer models, adavosertib monotherapy has demonstrated the ability to inhibit tumor growth. More importantly, when combined with chemotherapeutic agents such as Gemcitabine or Cisplatin, adavosertib significantly enhances DNA damage effects and overcomes tumor resistance to chemotherapy. Preclinical studies have confirmed that in p53-deficient cells, this combination strategy induces higher levels of abnormal mitosis and apoptosis, while p53 wild-type cells survive through intact G1/S arrest, providing a therapeutic window [53]. This selective killing mechanism based on p53 mutation status makes adavosertib a highly promising targeted drug for p53-mutant tumors.

ATR inhibitors (e.g., ceralasertib, elimusertib) are another class of drugs showing potent activity in p53-deficient tumors. ATR is a key kinase in the DNA damage repair pathway, primarily responsible for responding to replication stress. In p53-deficient tumor cells, due to the loss of the G1/S checkpoint, cells still enter S phase after DNA damage, leading to replication fork instability and increased replication stress [143]. ATR inhibitors block ATR-mediated DNA damage repair, preventing these cells from coping with replication fork collapse and ultimately causing cell death. Preclinical studies have shown that ceralasertib has significant antitumor activity in p53-mutant triple-negative breast cancer models [144]. Notably, the combination of ceralasertib with the PARP inhibitor Olaparib exhibits a strong synergistic effect. PARP inhibitors block single-strand DNA damage repair, leading to replication fork stalling, while ATR inhibitors further block repair, and their combination induces synthetic lethality in p53-mutant tumors [145]. This synergistic effect has been validated in p53-mutant triple-negative breast cancer models, providing new strategies for clinical treatment. Additionally, ATR inhibitors have shown monotherapy or combination therapy potential in p53-deficient ovarian cancer, lung cancer, and other models, with their mechanism relying on p53 mutation-induced defects in DNA damage repair, achieving specific killing of tumor cells.

### 7.3 Clinical translation and biomarker development

The clinical translation of synthetic lethality strategies in p53-deficient tumors faces multiple challenges. The primary challenge lies in the precise definition of p53 deficiency status, which involves not only detecting TP53 gene mutations but also distinguishing mutation types (e.g., missense vs. nonsense mutations) and assessing whether they lead to loss of function [31]. For example, in chronic lymphocytic leukemia (CLL), TP53 disruption (including mutations and 17p deletion) is the strongest predictor of chemotherapy resistance, directly guiding the selection of first-line treatment regimens [146]. However, tumor heterogeneity and activation of compensatory pathways further increase complexity. In neuroblastoma, p53 pathway inactivation is usually not caused by TP53 mutations but through changes in MDM2 or p14ARF expression, and the pRb pathway is often co-dysregulated, making inhibitors targeting a single pathway have limited efficacy, and even combination use may produce antagonistic effects [147]. Therefore, developing effective biomarkers for precise patient stratification is crucial. In addition to p53 mutation types, genomic instability scores such as homologous recombination deficiency scores and DNA damage response gene expression profiles are becoming important stratification tools [148]. In various cancers, TP53 mutations are associated with specific molecular features and clinical outcomes; for example, in endometrial cancer, abnormal p53 expression is associated with a specific immune microenvironment, potentially affecting immunotherapy response [149]. The integrated application of these biomarkers can help screen patient populations most likely to benefit from synthetic lethality therapies, providing direction for overcoming barriers to clinical translation.

## 8 Clinical translation challenges and future directions of p53-Targeted drug development

### 8.1 Mechanisms of drug resistance

The mechanisms by which tumor cells develop resistance to p53-targeted drugs are complex and diverse, with the activation of alternative signaling pathways being a key factor. For example, in dedifferentiated liposarcoma (DDLPS), MDM2, as a negative regulator of p53, can activate the p53 pathway upon inhibition, but tumor cells may bypass p53-dependent apoptosis programs by activating downstream signals such as PI3K/AKT or RAS/MAPK, thereby leading to drug resistance [149]. Additionally, secondary mutations or epigenetic changes in the p53 gene itself, such as DNA methylation, can significantly reduce drug sensitivity. In low-grade gliomas, TP53 mutation status is associated with resistance to multiple drugs; for instance, patients with TP53 mutations exhibit stronger resistance to dabrafenib and nutlin-3a (−) [150]. This indicates that the type of p53 mutation directly affects drug efficacy. Therefore, a key strategy to overcome resistance lies in combined targeting of multiple pathways. For example, in DDLPS, the combined use of the MDM2 inhibitor nutlin-3 with ferroptosis inducers Erastin or RSL3 can enhance cytotoxicity through synergistic effects, bypassing resistance issues from single-pathway inhibition [52]. This multi-target combination strategy offers new insights for addressing resistance to p53-targeted drugs.

### 8.2 Optimization of bioavailability and delivery systems

Many p53-targeted small-molecule drugs, such as APR-246, face pharmacokinetic challenges including poor water solubility and rapid metabolism, which limit their bioavailability and therapeutic efficacy in vivo. To overcome these obstacles, the development of novel drug delivery systems is crucial. Formulation technologies such as nanoparticles and liposomes can encapsulate drugs, improve their solubility and stability, and achieve passive targeting of tumor tissues through the enhanced permeability and retention (EPR) effect. Additionally, gene therapy vectors like Adeno-associated virus (AAV) and lentivirus have potential for delivering the p53 gene, but their inherent immunogenicity and potential integration risks need further reduction to ensure clinical safety. Targeted delivery systems, particularly antibody-drug conjugates (ADCs), by linking p53-targeted drugs to specific antibodies, can precisely deliver drugs to tumor sites, significantly increasing local drug concentrations while reducing systemic toxicity to normal tissues. Optimization of these delivery technologies is a key step in advancing p53-targeted drugs from the laboratory to clinical settings.

### 8.3 Biomarkers and patient stratification

The efficacy of p53-targeted drugs is highly dependent on individual patient characteristics, including the specific type of p53 mutation, tumor type, and composition of the tumor microenvironment (TME). Therefore, developing effective companion diagnostics for precise patient stratification is central to improving clinical trial success rates and treatment outcomes. For example, in low-grade gliomas, TP53 mutation status not only affects prognosis but also determines patient sensitivity to different drugs, suggesting that stratification based on p53 mutation type is necessary [151]. Liquid biopsy techniques, particularly detecting p53 mutations in circulating tumor DNA (ctDNA), provide a non-invasive means for real-time monitoring of treatment response and early detection of resistance. Furthermore, precise stratification based on p53 functional status (e.g., transcriptional activity, aggregation levels) is also crucial. For instance, in a functional constipation model, p53 activation is associated with apoptosis, while inhibition of p53 signaling protects cells [152]. Thus, assessing the activity status of p53 in tumor cells can help predict patient responses to p53-targeted drugs, enabling more precise treatment.

### 8.4 Optimization of combination therapy strategies

Combination regimens of p53-targeted drugs with chemotherapy, radiotherapy, immunotherapy, and other targeted drugs are being extensively explored to enhance efficacy and overcome resistance through synergistic effects. For example, in dedifferentiated liposarcoma (DDLPS), the combined use of the MDM2 inhibitor nutlin-3 with ferroptosis inducers Erastin or RSL3 significantly increases lipid peroxidation and cytotoxicity, demonstrating synergistic antitumor effects [153]. Additionally, in a cerebral ischemia reperfusion injury model, enhancing chaperone-mediated autophagy (CMA) can exert neuroprotective effects by inhibiting p53-mediated mitochondrial apoptosis, while the p53 activator nutlin-3 can reverse this protective effect [154]. These studies suggest that the timing and dosage of combination therapy are critical. For instance, using an MDM2 inhibitor to upregulate p53 activity first, followed by a ferroptosis inducer, may be more effective than simultaneous administration. Meanwhile, toxicity management of combination therapy needs optimization through preclinical models and early clinical trials to balance efficacy and safety, providing a basis for clinical translation.

### 8.5 Future research directions

Future development of p53-targeted drugs will move toward greater precision and personalization. First, the development of personalized drugs for rare p53 mutations (e.g., non-hotspot mutations) is an important direction, and the use of Artificial Intelligence (AI)-assisted drug design can accelerate this process by simulating the structure of mutant proteins to screen small molecules that can specifically restore their function. Second, exploring the non-cell-autonomous functions of p53 in the tumor immune microenvironment (TME), such as regulating the polarization of tumor-associated macrophages (TAMs), will provide new targets for immune combination therapy. p53 not only affects tumor cells themselves but also reshapes the TME through secreted factors or direct intercellular communication. Finally, advancing p53-targeted drugs from monotherapy to multimodal precision medicine requires integrating multidimensional data from genomics, proteomics, and metabolomics. For example, analyzing the metabolic characteristics of tumors can predict their sensitivity to ferroptosis inducers, thereby optimizing combination regimens [155]. This multi-omics integration strategy will help achieve truly individualized treatment and improve the clinical efficacy of p53-targeted drugs.

## 9 Conclusion

The development journey of p53-targeted drugs vividly demonstrates the arduous leap from basic scientific discovery to clinical translational application. Currently, small molecule reactivators represented by APR-246 and MDM2 inhibitors represented by Nutlin-3a have preliminarily validated the feasibility of the p53 pathway as an anti-tumor target, marking the transition of this field from “proof of concept” to the initial stage of “clinical practice.” However, we must clearly recognize that the achievements at this stage are far from the endpoint but rather the starting point of a new round of complex challenges.

From an expert perspective, the outcomes of different research pathways exhibit significant differences, requiring a careful balance of their value and limitations. Small molecule reactivators attempt to “repair” mutant p53 protein, with their core advantage lying in direct action on the target, but the major bottleneck is the diversity of mutation types—different mutation sites (e.g., R175H, R273H) show varying sensitivity to drugs and are prone to acquired resistance. In contrast, MDM2 inhibitors “release” wild-type p53 by blocking negative regulators, with clear efficacy, but their application is limited to patients retaining wild-type p53, and toxicity to normal tissues (such as thrombocytopenia) cannot be ignored. These two strategies are not mutually exclusive but complementary: for specific mutation types, combining reactivators with MDM2 inhibitors may achieve the dual effects of “activation” and “enhancement."

The emergence of emerging technologies provides new dimensions to break through current dilemmas. CRISPR gene editing technology can theoretically directly correct p53 mutations, but in vivo delivery efficiency, off-target effects, and ethical issues remain significant obstacles. PROTAC degradation technology takes a different approach by inducing ubiquitination and degradation of mutant p53 protein, eliminating its “dominant-negative effect” or “gain-of-function” activity, but its potential impact on normal p53 function requires rigorous evaluation. Immunotherapy strategies, such as p53 neoantigen vaccines or Bispecific T cell engagers, aim to transform mutant p53 into a target for immune attack, a highly innovative direction, but the immunosuppressive state of the tumor microenvironment may weaken its efficacy. These technological pathways are not isolated but should be viewed as a “toolbox”, future successful treatment strategies will likely need to dynamically combine these tools based on the patient's specific mutation type, tumor microenvironment characteristics, and immune status.

The core challenge of clinical translation lies in transforming laboratory “scientific miracles” into clinical “efficacy reality.” Drug resistance, poor delivery efficiency, and inadequate patient stratification are the three major “obstacles.” The mechanisms of drug resistance are complex, involving bypass pathway activation, epigenetic reprogramming, and tumor heterogeneity. Insufficient delivery efficiency requires the development of smarter nanocarriers or virus vectors to achieve precise tumor targeting. The root cause of inadequate patient stratification is our incomplete understanding of the correlation between p53 mutation profiles and clinical outcomes. Therefore, future research must shift from a “one-size-fits-all” treatment approach to a “Precision Medicine” model: establishing a molecular stratification system based on p53 mutation type, co-mutation profiles, and tumor immune microenvironment, thereby tailoring optimal combination therapy regimens for each patient.

In summary, p53-targeted therapy stands at a crossroads full of hope and challenges. True clinical breakthroughs will not come from the “solitary struggle” of a single technology but will inevitably be the product of deep multidisciplinary collaboration, molecular biology revealing resistance mechanisms, medicinal chemistry optimizing compound structures, nanotechnology solving delivery challenges, clinical oncology designing precise trials, and bioinformatics guiding patient stratification. Over the next decade, we should focus on: first, developing “specific” reactivators for different mutation types; second, exploring “rational combination” treatment strategies based on mechanistic understanding (e.g., p53 reactivators + immune checkpoint inhibitors); third, promoting real-time monitoring based on liquid biopsy to achieve dynamic treatment adjustments. Only in this way can p53, the “guardian of the genome,” truly transform into a “clinical weapon” against cancer.

## Generative AI use

During the preparation of this work, the authors did not used AI tool and take full responsibility for the content of the publication.

## Funding sources

This study was supported partly by grants from 10.13039/501100012166National Key Research and Development Program of China (2025YFE0126800); 10.13039/501100001809National Natural Science Foundation of China (82373348, 82572973), Open Fund Project of the NHC Key Laboratory of Diagnosis and Therapy of Gastrointestinal Tumor (NHCDP2025003), Jiading District Natural Science Research (JDKW-2025-0034), Changning District Science and Technology Committee (CNKW2024Y58), 10.13039/501100003392Natural Science Foundation of Fujian Province (2022J011427), the 905th Hospital of the Chinese 10.13039/501100017675People's Liberation Army Navy (2024J019), Taizhou Social Development Project Foundation (TS202309), Key Project Foundation of Taizhou School of Clinical Medicine, 10.13039/501100007289Nanjing Medical University (TZKY20240302), Taizhou Social Development Project Foundation (TS202309), Jiading District Science and Technology Committee (JDKW-2025-0034), and Jiading District Central Hospital (JDZXYY-2025-2).

## CRediT authorship contribution statement

**Hui-Deng Long:** Data curation. **Ning Zhang:** Data curation. **Wen-Er Wang:** Data curation. **Yu-Shui Ma:** Data curation. **Fu-Xing Liu:** Data curation. **Zi-Yu Chen:** Data curation. **Cui-Ni Lu:** Data curation. **Xiao-Feng Wang:** Data curation. **Ning Han:** Data curation. **Yue-Feng Cai:** Data curation. **Chun Yang:** Data curation. **Xiao-Mei Tang:** Data curation. **Hong Yu:** Data curation. **Hong Jiang:** Data curation. **Da Fu:** Conceptualization. **Kai-Jian Chu:** Conceptualization. **Wen-Guang Wu:** Conceptualization.

## Declaration of competing interest

The authors have declared no conflict of interest.

## Data Availability

Data will be made available on request.
